# Spin Hall voltages from a.c. and d.c. spin currents

**DOI:** 10.1038/ncomms4768

**Published:** 2014-04-30

**Authors:** Dahai Wei, Martin Obstbaum, Mirko Ribow, Christian H. Back, Georg Woltersdorf

**Affiliations:** 1 Institut für Experimentelle und Angewandte Physik, Universität Regensburg, Universitätsstraße 31, 93053 Regensburg, Germany; 2 Institut für Physik, Martin-Luther-Universität Halle, von-Danckelmann-Platz 3, 06120 Halle, Germany; 3 These authors contributed equally to this work

## Abstract

In spin electronics, the spin degree of freedom is used to transmit and store information. To this end the ability to create pure spin currents—that is, without net charge transfer—is essential. When the magnetization vector in a ferromagnet–normal metal junction is excited, the spin pumping effect leads to the injection of pure spin currents into the normal metal. The polarization of this spin current is time-dependent and contains a very small d.c. component. Here we show that the large a.c. component of the spin currents can be detected efficiently using the inverse spin Hall effect. The observed a.c.-inverse spin Hall voltages are one order of magnitude larger than the conventional d.c.-inverse spin Hall voltages measured on the same device. Our results demonstrate that ferromagnet–normal metal junctions are efficient sources of pure spin currents in the gigahertz frequency range.

## Abstract (web summary)

A spin current is injected from a ferromagnet into a nonmagnetic metal at magnetic resonance. Here, the authors show that this current has both a direct-current and a much larger alternating-current component, indicating that these structures could be useful for high-frequency spintronics.

## Graphical abstract

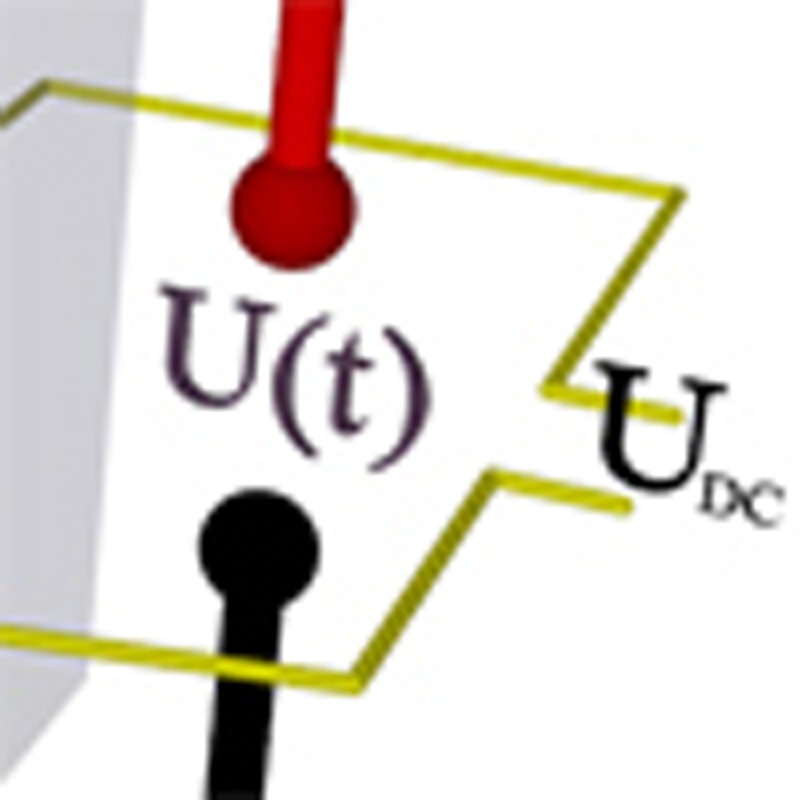

For spin electronic technology, the ability to create pure spin currents—that is, without net charge transfer—is essential. Spin pumping is the most popular approach to generate pure spin currents in metals^1^,^2^,^3^,^4^,^5^, semiconductors^6^,^7^, graphene^8^ and even organic materials^9^. When the magnetization vector in a ferromagnet (FM)–normal metal (NM) junction is excited at ferromagnetic resonance (FMR), spin pumping leads to the injection of pure spin currents in the NM. The polarization of this spin current is time-dependent^1^ and contains a very small d.c. component^10^, as illustrated in Fig. 1. Spin torque corresponding to the a.c. component has been observed by magneto-optical^11^ and X-ray methods^12^, while the spin accumulation because of the d.c. component was observed by light scattering^13^. Recently, also d.c. voltage signals in ferromagnetic insulator/ferromagnetic conductor bilayers have been interpreted as spin rectification in the ferromagnetic conductor material^14^. These experiments provide strong evidence for the presence of a large a.c. component of the spin current generated by spin pumping. The d.c. component of the injected spin current has been intensely studied in recent years and given rise to controversial discussions concerning the magnitude of the spin Hall angle, which is a material-dependent measure of the efficiency of spin-to-charge current conversion^15^,^16^. However, in contrast to the rather well-understood d.c. component^4^,^5^,^17^ the two orders of magnitude larger a.c. component has escaped experimental detection so far^18^.

The time dependence of the polarization of a spin current injected by spin pumping is related to the dyamics of the magnetization vector **m** and given by **σ**~**m** × *d***m**/*dt* (ref. 1) as illustrated in Fig. 1. The absorption of a spin current in a nonmagnetic metal with a finite spin Hall effect leads to an electric field **E** and is referred to as the inverse spin Hall effect (ISHE). The voltage *U*_ISHE_ transverse to the spin current **J**_**S**_ and spin polarization **σ** is:


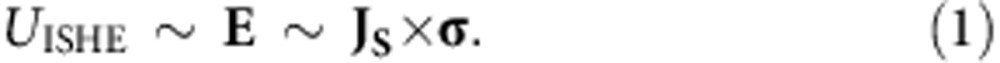


Therefore, the d.c. and a.c.-ISHE voltage components may be measured as shown in Fig. 1.

In the following, we demonstrate experimentally the presence of a large a.c. component in the ISHE voltage signal in NM/FM bilayers, where the a.c. spin current is generated by spin pumping at FMR. The magnitude of the a.c.-ISHE signal is measured as a function of frequency, angle and power. In addition, the d.c.- and a.c.-ISHE signals are measured in the same device in order to compare their relative amplitudes. The spectral shape, angular dependence, power scaling behaviour and absolute magnitude of the signals are in line with spin pumping and ISHE effects. Our results demonstrate that FM–NM junctions are very efficient sources of pure spin currents in the GHz frequency range and we believe that our result will stimulate the development of a.c. spintronics^18^,^19^.

## Results

### Experimental setup

The experimental configuration is shown in Fig. 2a, the NM–FM bilayer stripes are either integrated on top of the signal line or in the gap between the signal and ground lines of a grounded coplanar waveguide (CPW). In these two configurations, the magnetization in the FM is excited by an in-plane and out-of-plane microwave magnetic field **h**_rf_, respectively. The difficulty to detect the a.c.-ISHE signal lies in the ability to measure sub-mV GHz signals and isolate them from a large background signal caused by the excitation of FMR at the same frequency. As sketched in Fig. 2a, the microwave signal is transmitted from terminal 1 to terminal 2, where FMR can be measured inductively. In order to measure a.c.-ISHE signals, the NM–FM stripe is connected to a 50-Ω waveguide (terminal 3). In addition, the sample structure was designed as a transmission line (as microstrip for in-plane excitation and as CPW for out-of-plane excitation) such that the a.c.-ISHE voltage signal can propagate along the NM–FM stripe. The microwave signal isolation from terminal 1 to terminal 3 is only about 10 dB and is frequency-dependent (as shown in Supplementary Fig. 1) leading to a large crosstalk a.c. signal amplitude on terminal 3. This signal is 2 orders of magnitude larger than the expected a.c.-ISHE signal. In order to suppress the background signal, an additional reference signal is added in a power combiner where amplitude and phase can be adjusted to almost fully compensate the crosstalk signal. The expected ISHE signal has a magnitude in the mV range allowing for detection by a power meter (detection scheme 1) or by a rectifying diode and a lock-in amplifier (detection scheme 2). For lock-in detection the static magnetic field is modulated with an amplitude of 0.5 mT. The lock-in signal is converted into the a.c. voltage amplitude at terminal 3 using field integration and the power to voltage conversion characteristics of the Schottky detector.

### Dynamic properties

First the dynamic properties of the bilayer devices are studied by frequency-dependent FMR measurements. For these measurements, in-plane excitation is used and the magnetic field is applied along the *x* axis (*φ*_*H*_=90°). The results are summarized in Fig. 2b where a typical FMR spectrum obtained at a microwave frequency of 8 GHz is shown as the upper left inset. The resonance field *H*_r_ and line width Δ*H* are extracted from the spectra as a function of frequency. The frequency dependence of *H*_r_ can be well reproduced by a Kittel fit with effective magnetization *μ*_0_*M*_eff_=0.9 T. Δ*H* is strictly proportional to the microwave frequency, and the Gilbert damping constant determined from the slope of Δ*H*(*f*) is *α*=0.016, which is enhanced compared with *α*=0.008 obtained for a reference Ni_80_Fe_20_ layer, because of spin pumping^1^,^3^.

Typical signals of the a.c.-ISHE 
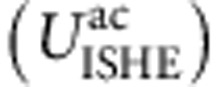
 measured at *φ*_*H*_=90° on a Pt/Ni_80_Fe_20_ stripe at 8 GHz using in-plane excitation are shown in Fig. 2c. The top spectrum (red line) is the amplitude of the a.c. voltage along the Ni_80_Fe_20_/Pt stripe measured directly with a microwave power meter (detection scheme 1), as outlined in Fig. 2a. At the resonance field, a step-like feature with an amplitude of 1 mV is observed. This signal is attributed to the a.c.-ISHE. The bottom spectrum (blue line) is the a.c.-ISHE signal measured by field modulation and lock-in amplification (detection scheme 2). This spectrum was converted into the voltage 
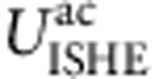
 by numerical integration. Line shape and amplitude are in agreement with the spectrum observed by the power meter; however, the signal-to-noise ratio is significantly improved. In the following, the line shape, frequency, power and angular dependence of the observed a.c.-ISHE signal will be examined in detail.

### Line shape

First, we would like to address the shape of the a.c.-ISHE signals. The signals we measure are a superposition of a field-independent microwave electric field (crosstalk between terminals 1 and 3), the actual a.c.-ISHE signal and a small inductive contribution. The antisymmetric line shape observed in Fig. 2c is a consequence of this superposition. Since the relative phase shift Φ_0_ between the electric crosstalk and the a.c.-ISHE signal is frequency- and sample-dependent, any line shape (symmetric to antisymmetric) can result. This is demonstrated by recording 
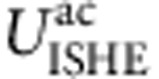
 spectra at frequencies between 3.5 and 10.5 GHz (from bottom to top) shown in Fig. 3a. As a function of microwave frequency, the a.c.-ISHE signals are observed at the negative and positive resonance fields of FMR, indicated by the grey line. The shapes of the resonance in 
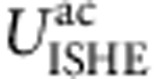
 can be peaks, dips or fully antisymmetric signals depending on the microwave frequency and device. The line shape of these spectra can be well explained by the superposition of two a.c. signals. A numerical simulation of the sum of 
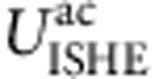
 and the background signal because of the crosstalk (*U*^bac^) for different phase shifts, Φ_0_ between these two signals^20^ is shown in the Supplementary Fig. 2.

### Power dependence

Figure 3b shows the rf-power dependence of *U*_ISHE_ at 6 GHz. *U*_ISHE_ is measured at *φ*_*H*_=90° and *φ*_*H*_=0°, respectively. The red dots and blue squares are for the a.c.- and d.c.-ISHE amplitudes, respectively. 
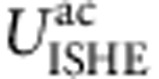
 and 
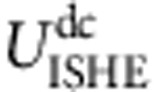
 are measured on different devices with in- and out-of-plane excitation fields, respectively. 
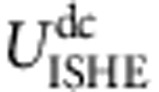
 is proportional to the rf-power *P*^5^,^21^, while 
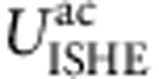
 scales with 
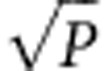
 as will be discussed below.

### Angular dependence

The angular dependence of 
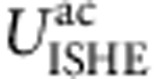
 measured at 6 GHz is shown in Fig. 3c,d. For this experiment, out-of-plane excitation is used and a rotatable magnetic field *H* is applied in the *x*–*y* plane; thus, the magnetic excitation and the spin pumping process do not depend on the in-plane field angle *φ*_*H*_. The spectra for *φ*_*H*_ between 90 and −90° (from top to bottom) are shown in Fig. 3c. The spectrum at *φ*_*H*_=90° (*H* applied along the stripe) shows a symmetric line shape, and its intensity decreases monotonically to zero when *φ*_*H*_ is 0° (*H* perpendicular to the stripe); for even smaller angles the signal reverses. The amplitude of 
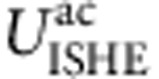
 as a function of *φ*_*H*_ is shown in Fig. 3d and can be well fitted to a sine function, as expected from Equation 1. At *φ*_*H*_=0°, since the a.c. spin current polarization **σ** is rotating in the *x*–*z* plane, the a.c.-ISHE voltage is generated along the *y* direction, leading to a vanishing voltage along the *x* direction (along the stripe). For the in-plane excitation, the measured a.c.-ISHE signals are symmetric under magnetic field reversal as expected from the symmetry of the susceptibility (*cf.* Fig. 3a Supplementary Fig. 3, and Supplementary Note 1).

### Signal amplitude

**Figure 1:**
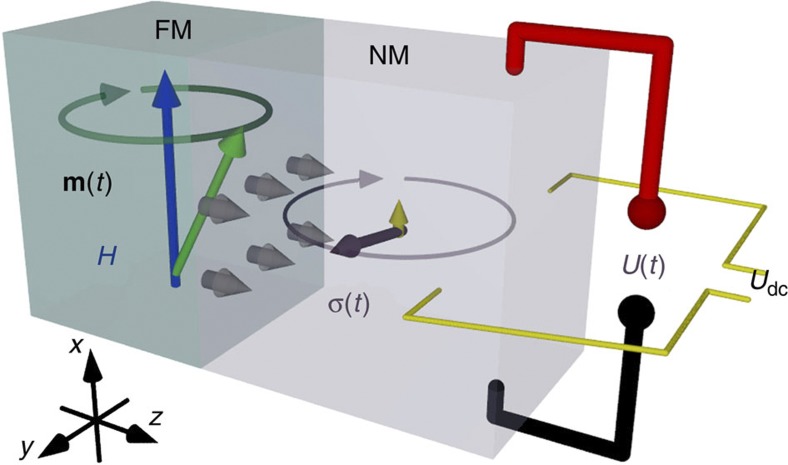
Spin pumping and ISHE voltage signal. A spin current is generated by spin pumping at the FM–NM interface (grey arrows). The time-dependent spin polarization of this current (indicated as purple arrow) rotates almost entirely in the *y*–*z* plane. The small time-averaged d.c. component (yellow arrow) appears along the *x* axis. Due to the inverse spin Hall effect both components lead to charge currents in NM and can be converted into a.c. and d.c. voltages by placing probes along the *x* and *y* directions, respectively.

In the following, we compare the amplitudes of the d.c.- and a.c.-ISHE signals. For the d.c.-ISHE measurements the voltage is measured by connecting a nanovoltmeter to terminal 3 of the sample. In Fig. 4a, the top (red) and bottom (black) spectra are the a.c.- and d.c.-ISHE voltages measured at 6 GHz with out-of-plane excitation. One can clearly see that the a.c.-ISHE signal is much larger than the d.c.-ISHE signal. For the measurement of the a.c.-ISHE the applied field is oriented at *φ*_*H*_=90°, while for the d.c.-ISHE *φ*_*H*_=0° is used (*cf.* Equation 1 and Fig. 1). For the measurements in Fig. 4a, we obtain a magnitude of 
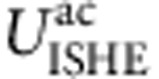
 and 
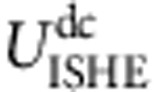
 of 120 and 10 μV, respectively.

Theoretically, one can derive the following expressions for the peak amplitudes^22^ (see Supplementary Note 1):


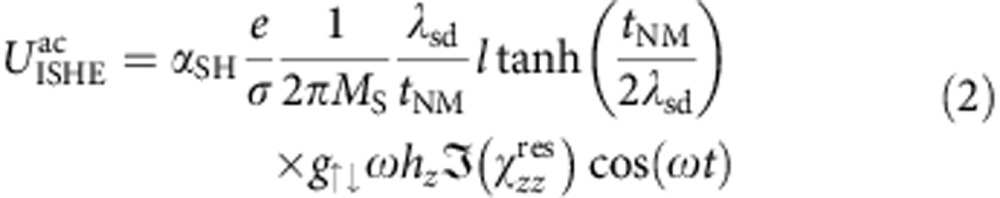



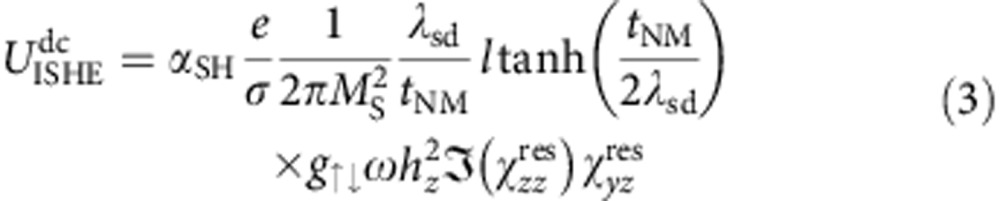


here *α*_SH_ and *λ*_sd_ are the spin Hall angle and spin diffusion length of NM, *l* is the length of the stripe and 
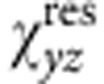
 and 
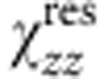
 are the in- and out-of-plane susceptibilities at FMR, respectively, while *h*_*z*_ is the magnetic out-of-plane microwave field amplitude. *g*_↑↓_ is the spin mixing conductance, *σ* is the conductivity of the bilayer and *t*_NM_ is the thickness of the NM (for example, Pt) layer. Since the d.c.-ISHE signal scales with 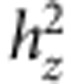, one expects 
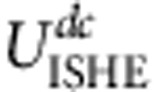
 to scale linearly with the microwave power *P*^5^,^21^, while for a.c.-ISHE a scaling behaviour with *P*^1/2^~*h*_*z*_ is expected (cf. Equation 2). This behaviour is perfectly reproduced in our experiment as shown in Fig. 3b. Furthermore, the expected ratio of the amplitudes of the a.c.- and d.c.-ISHE voltages is given by (see also Supplementary Note 1)


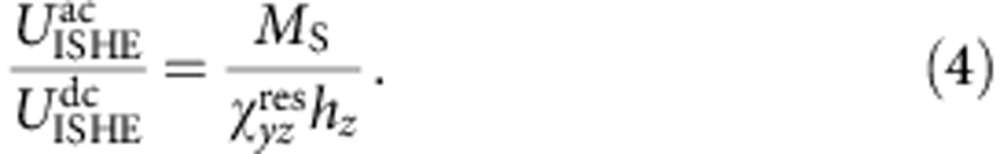


This ratio can be easily calculated for parameters that apply to the measurements shown in Fig. 3a: *f*=6 GHz (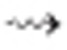
*H*_r_=45 mT), using *μ*_0_*M*_S_=0.9 T, 
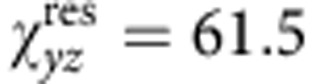
 and *μ*_0_*h*_*z*_=0.3 mT^22^ results in 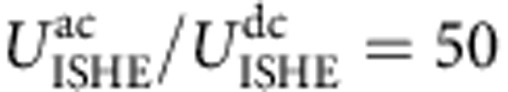. Experimentally, we only observe 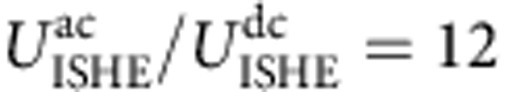; however, one needs to consider the poor transmission of the rf signal into the 50-Ω terminal. In the case of out-of-plane excitation, the bilayer stripe in the gap of the CPW can be considered as a waveguide with a characteristic impedance of 250 Ω. The resistance mismatch between this waveguide and the 50-Ω terminal leads to a transmission of only 33% of the signal as can be calculated from the voltage standing wave ratio 
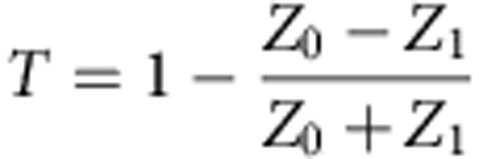
 (see Supplementary Note 2). This implies that the 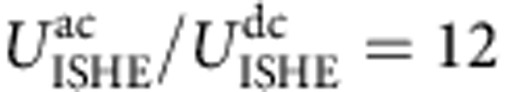 is in fact three times larger at the sample. Using published values for the spin Hall angle *α*_SH_=0.12 (refs 22, 23) and *λ*_sd_=1.4 nm^23^,^24^, the ISHE voltages at resonance (6 GHz) can be calculated as 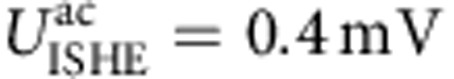 and 
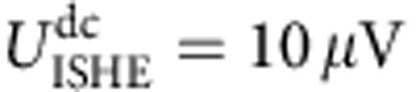
, respectively. Note that for the sake of simplicity no backflow correction as suggested in Jiao and Bauer^18^ is considered here. The observed 
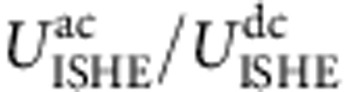
 ratio and the absolute amplitude (*cf.* Fig. 4a) is in agreement with theory^18^ and previous d.c.-ISHE experiments. A similar analysis can be performed with the signal amplitudes shown in Fig. 3a. For example, for FMR at 10 GHz one obtains the following parameters: 
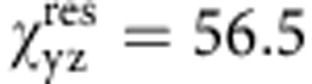
 (because of the in-plane excitation 
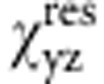
 has to be used instead of 
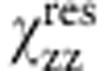
) and *μ*_0_*h*_*y*_=0.27 mT. From this and Equation 2, a peak-to-peak amplitude of 4.2 mV is expected. In addition, the waveguide properties of the Ni_80_Fe_20_/Pt stack on top of the gold waveguide need to be considered. As discussed in Supplementary Note 2, this configuration is equivalent to a microstrip with a characteristic impedance *Z*_0_=480 Ω. One expects a transmission of only 18% into *Z*_1_=50 Ω using the voltage standing wave ratio. Therefore, the expected amplitude is 0.7 mV. Experimentally, we find an amplitude of 0.5 mV in excellent agreement with theory (see Fig. 3a).

## Discussion

The measured a.c. signals may also be generated by parasitic mechanisms instead of ISHE. These are (i) inductive coupling of the magnetization with the conducting wire loop used for signal detection and (ii) anisotropic magnetoresistance (AMR). The magnitude of both of these effects will be addressed in the following.

**Figure 2:**
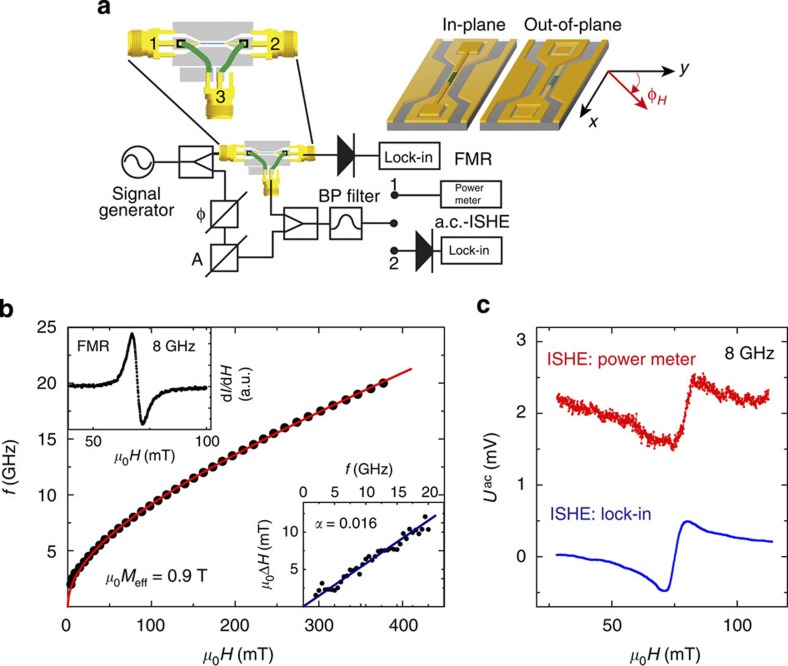
Detection of a.c. spin currents by ISHE. (**a**) Layout of the measurement configuration. The microwave signal is split into a part that excites the sample and a reference arm where amplitude and phase can be adjusted independently. The signal on terminal 2 is used for inductive FMR measurements, while the signal on terminal 3 originates from a.c.-ISHE. This signal is either measured using a power meter or a lock-in amplifier. In-plane rf excitation (*h*_*y*_) is used when the bilayer stripe is placed on top of the signal line of the CPW, while placing the bilayer in the gap between signal line and ground planes leads to an out-of-plane excitation field (*h*_*z*_). (**b**) FMR resonance field as a function of microwave frequency. The upper left inset shows a typical FMR spectrum of the Pt/Ni_80_Fe_20_ bilayer measured at 8 GHz, the bottom right inset shows the frequency dependence of the resonance line width *μ*_0_Δ*H*. (**c**) a.c.-ISHE spectra at 8 GHz measured using a power meter (red) and using field modulation and lock-in amplification (blue).

The exclusion of an inductive signal component in the presumed ISHE signal cannot be based on angular or rf-power dependency since the amount of out-of-plane magnetic flux generated by the in-plane component of the magnetization has the same angular and power dependence as the ISHE signal^25^, as illustrated in Supplementary Fig. 4. For this reason, we use a series of different conducting materials with different spin Hall angles to quantify the importance of inductive coupling in our experiments. In Fig. 4b,c we show the a.c. voltage signals generated at 8 GHz by Pt/Ni_80_Fe_20_, Au/Ni_80_Fe_20_, Cu/Ni_80_Fe_20_ and Al/Ni_80_Fe_20_ bilayers with identical thicknesses (only the NM=Cu layer has a thickness of 20 nm). The experiments are performed for both in-plane and out-of-plane configurations (*cf.* Fig. 2b). The scale bar for the out-of-plane data in Fig. 4c was chosen such that the signal amplitude for the Pt/Ni_80_Fe_20_ measurement is equal to the in-plane case. From the fact that the signal for Au/Ni_80_Fe_20_ (90 μV) is about 10% of the Pt/Ni_80_Fe_20_ signal (648 μV) it becomes clear that the inductive contribution must be less than 10% for the Pt/Ni_80_Fe_20_. For further details we refer to Supplementary Note 3 and Supplementary Fig. 4. For Al and Cu, it is well accepted that the spin Hall effect is very small because of the weak spin–orbit interaction^26^,^27^. Therefore, our conclusion is further corroborated by additional experiments on Cu/Ni_80_Fe_20_ and Al/Ni_80_Fe_20_ bilayers as shown in Fig. 4b,c where in agreement with smaller spin Hall angles in these materials a similarly low signal magnitude was found. It is also obvious that the signal amplitudes for these different samples are very reproducible even when a different excitation or coupling geometry is used as demonstrated by comparing Fig. 4b,c. Furthermore, if the NM layer thickness is doubled, the inductive signal amplitude is also doubled (*cf.* Supplementary Fig. 5). Samples NM=10 nm Cu and NM=10 nm Pt have almost identical resistances of 1.6 and 1.7 kΩ, respectively. Therefore, comparing the magnitude of the a.c. voltage generated in these two samples provides the most accurate estimate of the inductive contribution. From Fig. 4c and Supplementary Fig. 5 we have 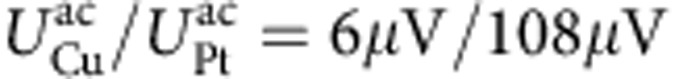 and one can conclude that the inductive coupling contribution is only 5% in the Pt/NiFe bilayers.

A possible AMR contribution can be determined by examining the angular dependence of the signal measured for Al/Ni_80_Fe_20_ (where no measurable a.c.-ISHE signal is expected). In the vicinity of *φ*_*H*_=90° the precessing magnetization leads to a small 2*ω* variation of the wire resistance because of AMR. This time-dependent resistance mixes with the inductively or capacitively coupled microwave current in the metallic bilayer stack oscillating at *ω*. The corresponding a.c. voltage is given by *U*_AMR_=*I*(*ω*)*R(2*ω*) with mixing products oscillating at *ω* and 3*ω*. Using a band pass filter with a pass band centred at *ω*, only the 3*ω* contribution can be suppressed. For the given excitation amplitude, wire resistance and AMR amplitude, the a.c.-AMR voltage at *ω* can be estimated to have a magnitude of less than 1 μV for the Al/NiFe sample. As derived in equation 4 of Mecking *et al.*^28^ the dominating 2*ω* component of the resistance follows a cos(2*φ*_*H*_) dependence and vanishes at *φ*_*H*_=45°. We experimentally verify the insignificance of the AMR contribution by comparing signals at *φ*_*H*_=90° and *φ*_*H*_=45°. From Supplementary Fig. 6 one sees that the signal amplitude follows the cos(*φ*_*H*_) dependence that is consistent with inductive coupling.

The capping layer material dependence, the angular dependence, power dependence, line shape and magnitude of the signal are in line with the theory of a.c.-ISHE and strongly indicate that the a.c. signals measured for Pt/Ni_80_Fe_20_ bilayers are indeed a consequence of the spin currents generated by spin pumping and detected by the ISHE.

In summary, we demostrated the presence of large a.c.-ISHE signals because of spin pumping at FMR with a.c.-ISHE signals reaching amplitudes of up to 1.5 mV. The direct comparison of the a.c.- and d.c.-ISHE voltage on the same device for out-of-plane excitation shows that 
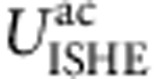
 is ~12 times larger than 
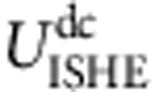
, despite the fact that our experiment can only detect 33% of the a.c.-ISHE signal. The large a.c.-ISHE voltages indicate the presence of large rf spin currents in agreement with the theory of spin pumping. Such spin currents and their detection via ISHE may prove very useful for the development of future a.c. spintronic devices^19^.

## Methods

### Sample fabrication

The bilayer stripes are prepared by electron beam lithography, magnetron sputter deposition and lift-off techniques on semi-insulating GaAs substrates. Subsequently, the CPW and the electrical contacts are fabricated by optical and electron beam lithography using gold metallization. All FM and NM layers in this manuscript have a thickness of 10 nm. Only the NM=Cu layer used for in-plane excitation in Fig. 4b has a thickness of 20 nm. A thick alumina layer (50 nm Al_2_O_3_) deposited by atomic layer deposition is used to insulate the NM–FM bilayer stripes and the contact electrodes from the CPW. In all experiments the stripes are 5 μm wide and 400 μm long.

### Electrical sample properties

**Figure 3:**
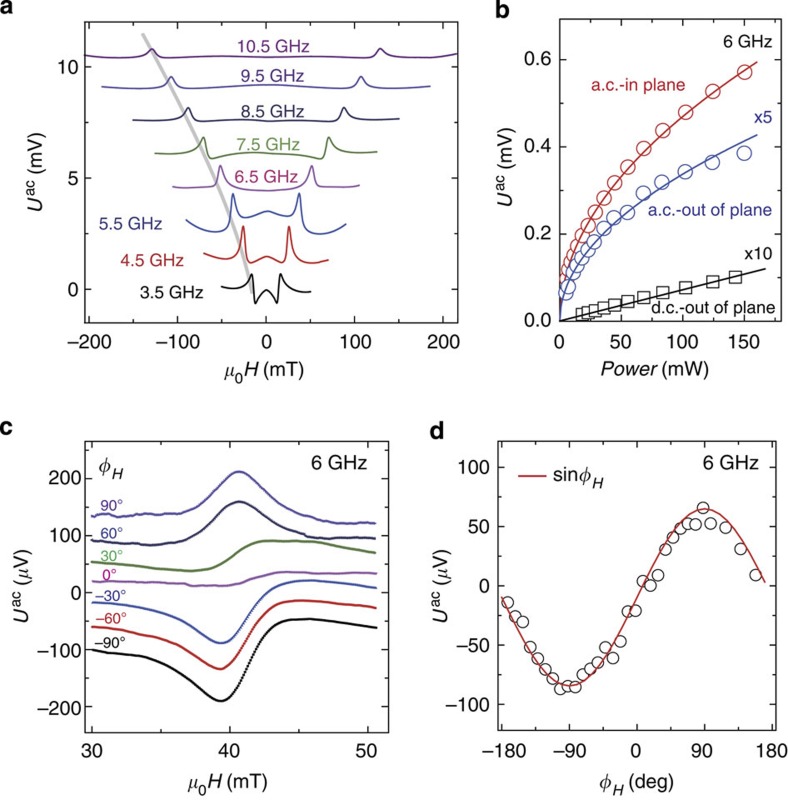
The frequency, power and angular dependence of the a.c.-ISHE signals. (**a**) The a.c.-ISHE voltages measured by a lock-in amplifier at microwave frequencies from 3.5 to 10.5 GHz using in-plane excitation. (**b**) The microwave power (*P*) dependence of 
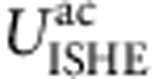
 (in-plane and out-of-plane excitation) and 
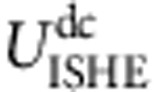
 (out-of-plane excitation) at 6 GHz, for comparison the a.c.- and d.c.- signals measured with out-of-plane excitation are multiplied by 5 and 10, respectively. The solid lines are fits to 
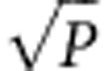
 and *P* for a.c.- and d.c.-ISHE, respectively. (**c**) a.c.-ISHE measured at 6 GHz using out-of-plane excitation with different field angles *φ*_*H*_ from −90° to 90°. (**d**) Angular dependence of the amplitude of 
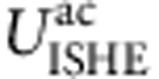
. Note that for in-plane excitation one finds an even symmetry of the a.c.-ISHE signal with respect to the direction of the applied field (**a**), while for out-of-plane excitation one finds an odd symmetry (**c**). This behaviour is expected from the symmetry of the susceptibility.

**Figure 4:**
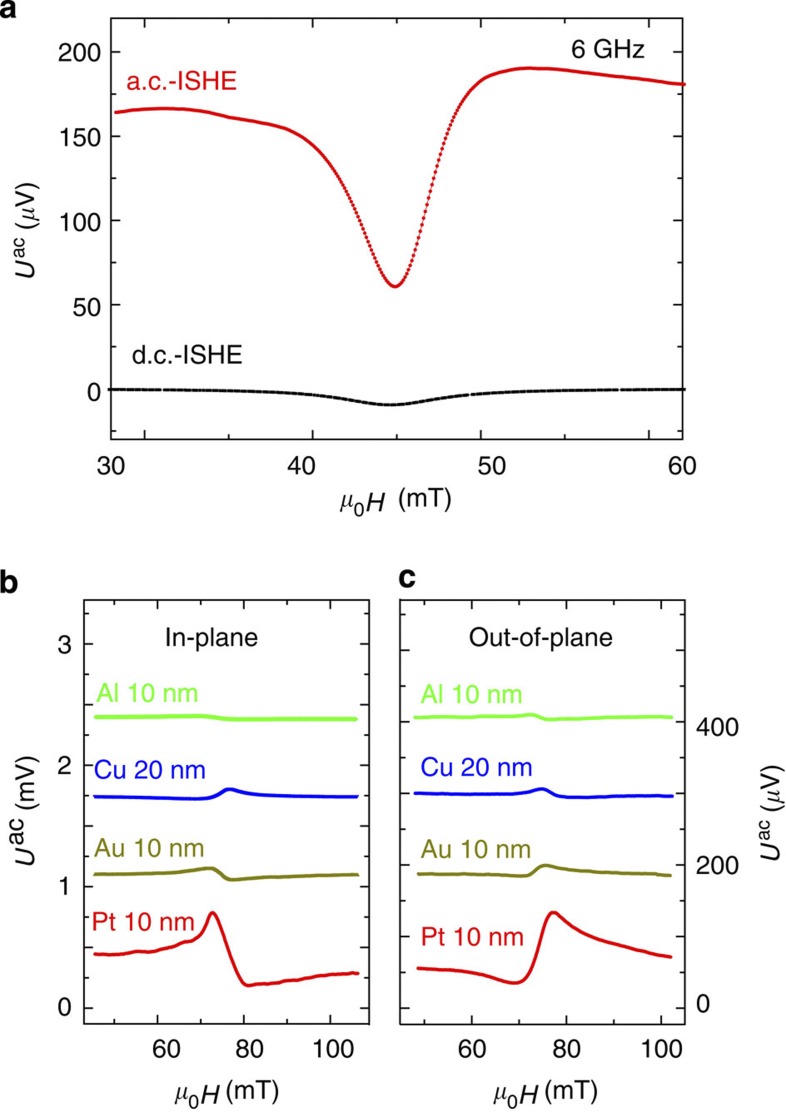
Comparison of the a.c.- and d.c.-ISHE amplitude and material dependence. (**a**) Comparison of the a.c.- and d.c.-ISHE voltages for the same device measured at 6 GHz in the out-of-plane excitation configuration. The a.c.-ISHE voltage is ~12 times larger than the d.c. one. (**b**,**c**) Comparison of the a.c.-ISHE signals for Pt/Ni_80_Fe_20_, Au/Ni_80_Fe_20_, Cu/Ni_80_Fe_20_ and Al/Ni_80_Fe_20_ measured at 8 GHz. (**b**) Shows data for samples with in-plane excitation while (**c**) shows the corresponding measurements with out-of-plane excitation. All NM and FM layers have a thickness of 10 nm. Only for NM=Cu the NM layer is 20 nm.

The electrical resistance of the NM–FM bilayer stripes are 1.6, 0.7, 0.7 and 2.9 kΩ for Pt/Ni_80_Fe_20_, Au/Ni_80_Fe_20_, Cu/Ni_80_Fe_20_ and Al/Ni_80_Fe_20_, respectively. Note that the Cu/Ni_80_Fe_20_ bilayer is 20-nm thick. A 10-nm-thick Ni_80_Fe_20_ single layer has a resistance of 4.2 kΩ. All measurements are performed at room temperature. The input microwave power was nominally constant and fixed at 320 mW (25 dBm) for all experiments except for the measurements shown in Figure 3b.

## 

## Author contributions

G.W. and C.H.B. designed and supervised the experiments. D.W., M.O. and G.W. prepared the experimental setup. D.W. and M.O. performed the experiments. The samples were prepared by M.O. and M.R. D.W. and M.O. carried out the data analysis. D.W., M.O., C.H.B. and G.W. wrote the paper. All authors analysed the data, discussed the results and commented on the manuscript.

## Additional information

**How to cite this article:** Wei, D. *et al.* Spin Hall voltages from a.c. and d.c. spin currents. *Nat. Commun.* 5:3768 doi: 10.1038/ncomms4768 (2014).

## Supplementary Material

Supplementary Information Supplementary Figures 1-6, Supplementary Notes 1-3 and Supplementary References
