# Supplementary material for: Spin Hall voltages from a.c. and d.c. spin currents
Source: Nat Commun. 2014 Apr 30;5:3768. doi: 10.1038/ncomms4768 (PMC4015325; doi:10.1038/ncomms4768)
Supplement: Supplementary Information — Supplementary Figures 1-6, Supplementary Notes 1-3 and Supplementary References [file ncomms4768-s1.pdf]

I. SUPPLEMENTARY FIGURES

Supplementary Figure 1

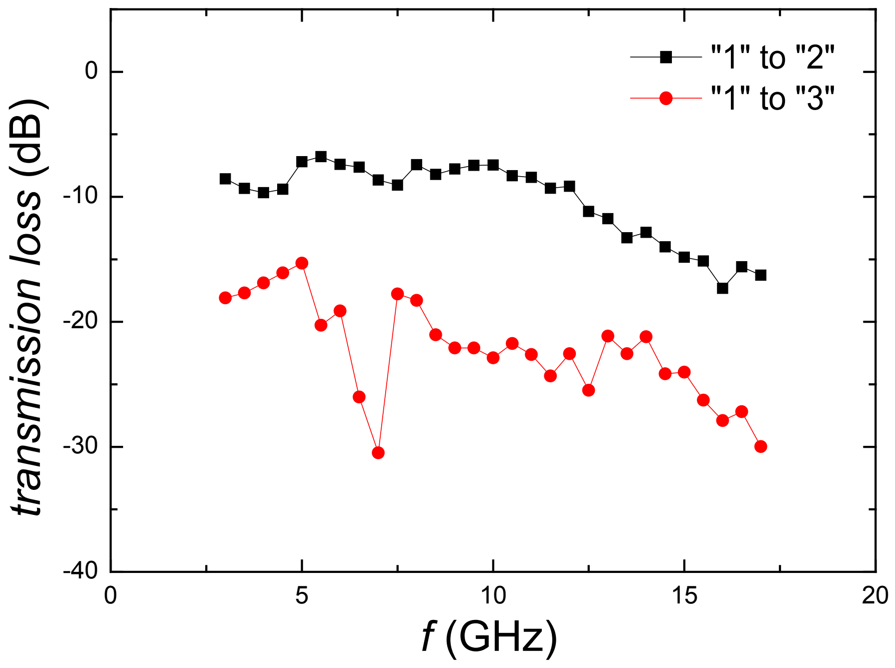

Supplementary Figure 1. **Frequency dependence of the microwave transmission between terminals 1 (input), terminal 2 (output) and 3 (ac- pickup).** In addition to the partial transmission caused by resistance mismatch there are additional ohmic losses and losses caused by transmitting ac signals across bonding wires. The black squares show the transmission characteristics from input (terminal 1) to output (terminal 2). The loss is typically around -10 dB and has been used to estimate the actual microwave field as at the $Ni_{80}Fe_{20}/Pt$ stack. In addition the red dots show the transmission from input (terminal 1) to the ac pick-up (terminal 3). The transmission is approximately -20 dB and causes the cross talk amplitude U^{bac} on terminal 3.

Supplementary Figure 2

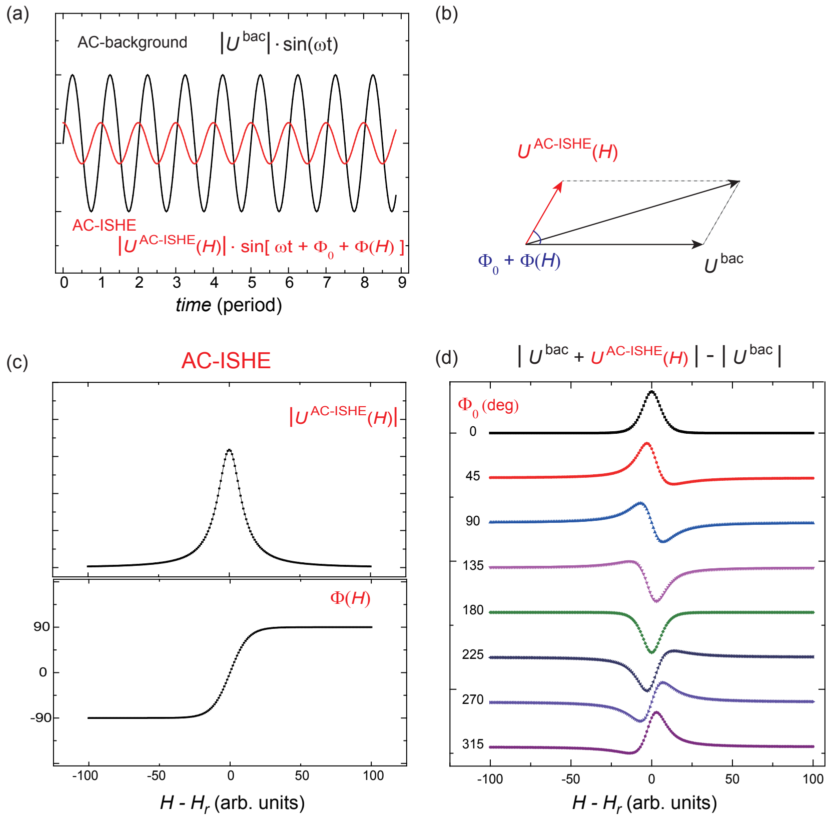

Supplementary Figure 2. **Superposition of ac-ISHE U^{ac}_{ISHE} and ac-cross talk U^{bac}.** (a) The ac-ISHE voltage U^{ac}_{ISHE} (red curves) is added to a background ac-cross talk voltage U^{bac} (black curve). U^{ac}_{ISHE} has a phase shift of $\Phi_0 + \Phi(H)$ with respect to U^{bac}, where Φ_0 and $\Phi(H)$ are initial and field dependent parts. Φ_0 is sample as well as frequency dependent. (b) The signal detected by the diode is the amplitude of the vector sum of U^{ac}_{ISHE} and U^{bac} in the complex plane. This quantity is therefore very sensitive to the relative phase shift between U^{ac}_{ISHE} and U^{bac}. (c) Field dependence of the amplitude and phase of U^{ac}_{ISHE} around the resonance field (H_{res}). The amplitude is given by the square root of a Lorentzian line shape as expressed in Eq. 11 of Supplementary Note 1. The phase $\Phi(H)$ experiences a total phase shift of π. (d) Simulated ac-ISHE spectra ($|\ U^{bac} + U^{ac}_{ISHE}\ | - |\ U^{bac}\ |$) for various values of Φ_0. The shapes of all experimental spectra can be well reproduced by a suitable choice of Φ_0. When $\Phi_0 = 0°$, the peak like shape (for example the spectrum at 9.5 GHz in Fig. 2 (a)) is expected; while $\Phi_0 = 90°$, the line shape is antisymmetric, e.g. as in the experimental spectrum measured at 7.5 GHz in Fig. 2(a).

Supplementary Figure 3

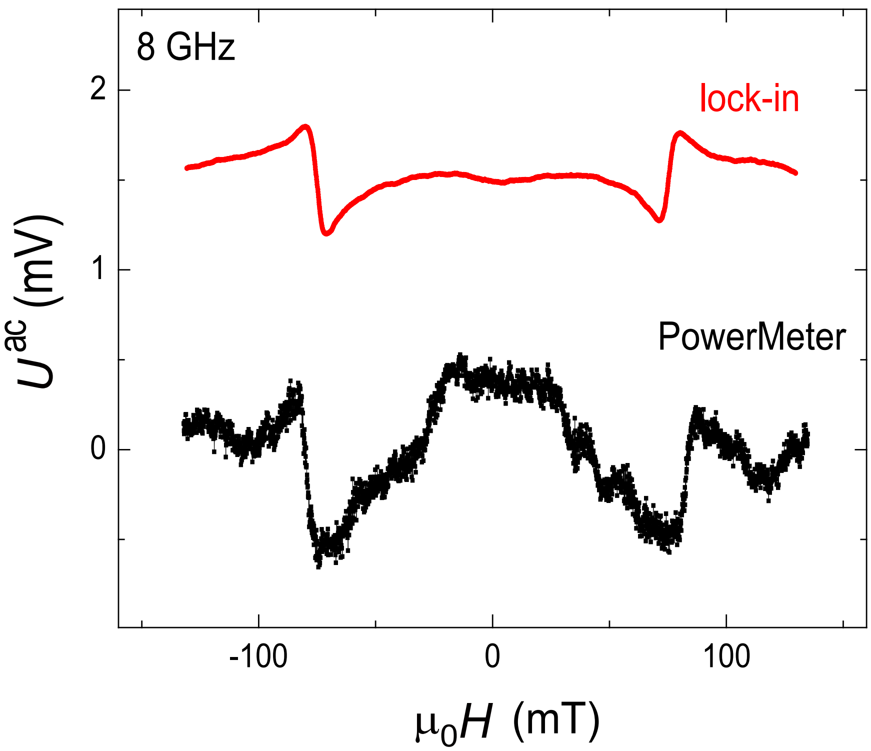

Supplementary Figure 3. **Comparison of $U^{\rm ac}_{\rm ISHE}$ measured using a simple power meter and lock-in detection for in-plane excitation.** The bottom black curve is the ac-ISHE signal obtained using a microwave power meter, while the upper red curve is measured using field modulation and lock-in detection. One should point out that the $U^{\rm ac}_{\rm ISHE}$ signal is symmetric with respect to field inversion. As expressed in Eq.7 of Supplementary Note 1, the phase of ac spin current is given by $(M_{\rm S} m_z)$, where m_z equals $\chi_{zz}h_z$ and $\chi_{zy}h_y$ for the in-plane and out-of-plane excitation, respectively. Under field inversion $M_{\rm S}$ and χ_{zy} change sign, while the corresponding susceptibility element χ_{zz} does not, thus from theory $U^{\rm ac}_{\rm ISHE}$ is expected to be symmetric for in-plane excitation (in agreement with the experiment). On the other hand for out-of-plane excitation the sign reversal of χ_{zy} leads to an antisymmetric behavior under field inversion (cf. experimental result in Fig. 3(c) and (d) of the manuscript).

Supplementary Figure 4

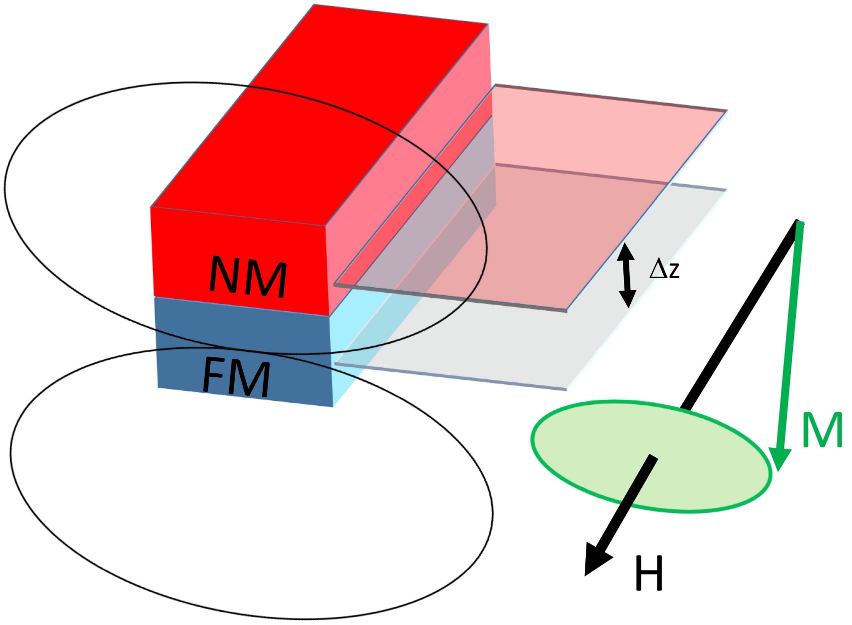

Supplementary Figure 4. **Inductive coupling mechanism.** Ac-signals can also be generated when the height of the conducting loop shifts by ΔZ with respect to the center of the precessing magnetic moment. When the conducting loop is shifted magnetic flux that is generated by the precessing magnetization penetrates the conducting loop and generates an ac-voltage signal as indicated by the black ellipses (Faraday's law).

Supplementary Figure 5

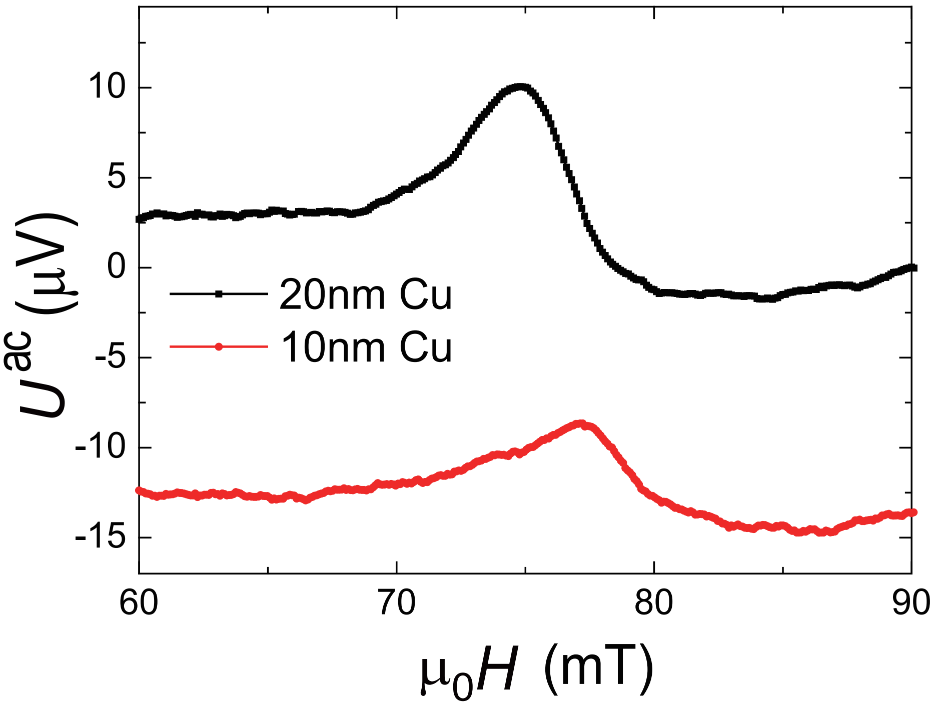

Supplementary Figure 5. **NM thickness dependence of the parasitic ac-signal for the Cu/$Ni_{80}Fe_{20}$ bilayer.** Ac-signals measured at $\phi_H = 90°$ with out-of-plane excitation geometry at a microwave frequency of 8 GHz. The increase amplitude for larger NM thickness is consistent with the indcuctive mechanism sketched in Supplementary Fig. 4.

Supplementary Figure 6

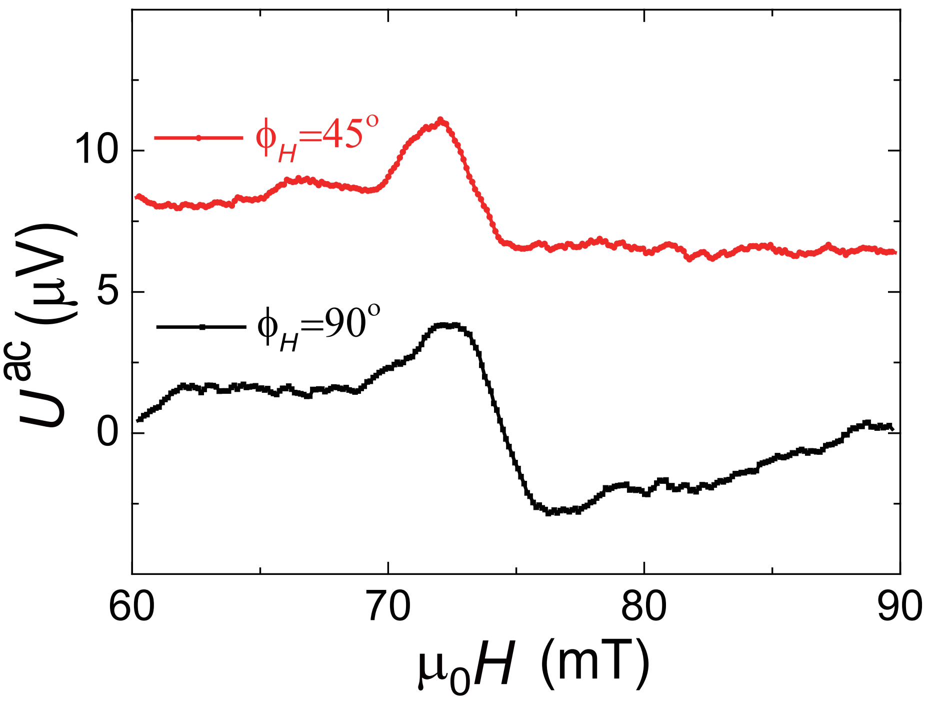

Supplementary Figure 6. **Angular dependence of the parasitic ac-signal for the Al/Ni$_{80}$Fe$_{20}$ bilayer.** Ac-signals measured at $\phi_H = 90°$ and $\phi_H = 45°$ for a Al/Ni$_{80}$Fe$_{20}$ bilayer sample with out-of-plane excitation geometry at a microwave frequency of 8 GHz. The amplitude variation is consistent with a $\sin(\phi_H)$ dependence. Therefore a significant AMR contribution to the ac-signal is experimentally excluded.

II. SUPPLEMENTARY NOTES

Supplementary Note 1

Derivation of signal amplitudes for ac- and dc- ISHE components. In the case of spin pumping due to FMR, the spin currents are generated from the precession of the magnetization which is described by the Landau-Lifshitz-Gilbert equation:

$$\frac{d\mathbf{M}}{dt} = -\mu_0\gamma(\mathbf{M}\times\mathbf{H}_{\text{eff}}) + \frac{\alpha}{M_{\text{S}}}(\mathbf{M}\times\frac{d\mathbf{M}}{dt}) \tag{1}$$

where $\mathbf{M}$ and $\mathbf{H}_{\text{eff}}$ are magnetization and effective field, γ is the electron gyromagnetic ratio, α is the damping constant. The magnetization $\mathbf{M}$ can be expressed as:

$$\mathbf{M} = \frac{1}{2}(2M_{\text{S}}, m_y e^{i\omega t} + m_y^* e^{-i\omega t}, m_z e^{i\omega t} + m_z^* e^{-i\omega t}) \tag{2}$$

and

$$\begin{aligned} m_y &= \chi_{yy}h_y + \chi_{yz}h_z \\ m_z &= \chi_{zy}h_y + \chi_{zz}h_z \end{aligned} \tag{3}$$

in which χ_{yy}, χ_{yz}, χ_{zy}, χ_{zz} are susceptibility tensor elements, and h_y, h_z are the in and out-of-plane rf excitation fields. The spin current density generated by spin pumping is given by [1, 2]:

$$J_{\text{S}}\boldsymbol{\sigma} = \frac{\hbar}{4\pi}g_{\uparrow\downarrow}\hat{\mathbf{m}}\times\frac{d\hat{\mathbf{m}}}{dt} \tag{4}$$

Where $\hat{\mathbf{m}}$ is $\mathbf{M}/M_{\text{S}}$, and $g_{\uparrow\downarrow}$ is the spin mixing conductance.

Ac- and dc- charge currents generated from a spin current via the ISHE can be described by the following formula:

$$\mathbf{J}_{\text{C}} = \alpha_{\text{SH}}\frac{2\text{e}}{\hbar}J_{\text{S}}\widehat{\mathbf{e}}_z\times\boldsymbol{\sigma} \tag{5}$$

where α_{SH} is the spin Hall angle, $\widehat{\mathbf{e}}_z$ indicates that the spin current propagates along the z direction, $\boldsymbol{\sigma}$ is the polarization vector given by Eq. 4. The dc spin current is given by the time average of $J_{\text{S}}\boldsymbol{\sigma}$ which is polarized in the x direction and given by:

$$[J_{\text{S}}\overrightarrow{\sigma}]_t = \frac{\hbar\omega g_{\uparrow\downarrow}}{2\pi\text{M}_{\text{S}}^2}\Im(m_y^* m_z)\widehat{\mathbf{e}}_x. \tag{6}$$

While the ac spin current causing the ac ISHE signal detected in the x direction is given by:

$$J_{\mathrm{S}}\sigma_y = \frac{\hbar\omega g_{\uparrow\downarrow}}{2\pi \mathrm{M}_{\mathrm{S}}^2}(M_{\mathrm{S}}m_z)\cos(\omega t)\widehat{\mathbf{e}}_y \tag{7}$$

From Eqs. 6 and 7, the ratio of the ac and ac spin current amplitudes is found as

$$\frac{J_{\mathrm{S}}\sigma_y}{[J_{\mathrm{S}}\boldsymbol{\sigma}]_t} = \frac{M_{\mathrm{S}}m_z}{\Im(m_y^* m_z)} \tag{8}$$

In the case of out-of-plane excitation, i.e., $h_{\mathrm{rf}} = h_z$, Eqs. 7 and 8 can be simplified as:

$$J_{\mathrm{S}}\sigma_y = \frac{\hbar\omega g_{\uparrow\downarrow}}{2\pi M_{\mathrm{S}}^2} M_{\mathrm{S}} h_z \chi_{zz}\cos(\omega t)\widehat{\mathbf{e}}_y \tag{9}$$

$$[J_{\mathrm{S}}\boldsymbol{\sigma}]_t = \frac{\hbar\omega g_{\uparrow\downarrow}}{2\pi M_{\mathrm{S}}^2} h_z^2 \Im(\chi_{zz})\chi_{yz}\widehat{\mathbf{e}}_x \tag{10}$$

The different microwave power dependence of the dc and ac ISHE signals can be understood from Eqs. 9 and 10. The magnitude of the dc spin current is proportional to the square of the excitation field h_z^2 (proportional to the microwave power P). On the other hand the magnitude of the ac spin current, it proportional to h_z (or the square root of microwave power $\sqrt{P}$).

By combining the values of susceptibility tensor elements at FMR, and neglecting the small $(\Delta H/H_{\mathrm{r}})$ term, the ac- and dc-ISHE voltages can be expressed as follows:

$$U_{\mathrm{ISHE}}^{\mathrm{ac}} = \alpha_{\mathrm{SH}}\frac{e}{\sigma}\frac{1}{2\pi \mathrm{M}_{\mathrm{S}}}\frac{\lambda_{\mathrm{sd}}}{t_{\mathrm{NM}}} l \tanh(\frac{t_{\mathrm{NM}}}{2\lambda_{\mathrm{sd}}}) g_{\uparrow\downarrow}\omega h_z \Im(\chi_{zz}^{\mathrm{res}})\cos(\omega t) \times \frac{\Delta H}{\sqrt{(H-H_{\mathrm{r}})^2 + (\Delta H)^2}}, \tag{11}$$

$$U_{\mathrm{ISHE}}^{\mathrm{dc}} = \alpha_{\mathrm{SH}}\frac{e}{\sigma}\frac{1}{2\pi \mathrm{M}_{\mathrm{S}}^2}\frac{\lambda_{\mathrm{sd}}}{t_{\mathrm{NM}}} l \tanh(\frac{t_{\mathrm{NM}}}{2\lambda_{\mathrm{sd}}}) g_{\uparrow\downarrow}\omega h_z^2 \Im(\chi_{zz}^{\mathrm{res}})\chi_{yz}^{\mathrm{res}} \times \frac{(\Delta H)^2}{(H-H_{\mathrm{r}})^2 + (\Delta H)^2}, \tag{12}$$

where λ_{sd} is the spin diffusion length of the normal metal, l is the length of the bilayer stripe, t_{NM} is the thickness of the normal metal, σ is the conductivity of the bilayer stripe, and χ_{zz}^{res} and χ_{yz}^{res} are the susceptibility tensor elements at the resonance field. At the resonance field, the ratio of amplitude of the ac- and dc- ISHE voltages can be simplified to

$$\frac{U_{\mathrm{ISHE}}^{\mathrm{ac}}}{U_{\mathrm{ISHE}}^{\mathrm{dc}}} = \frac{M_{\mathrm{S}}}{\chi_{yz}^{\mathrm{res}} h_z}. \tag{13}$$

Supplementary Note 2

Transmission efficiency of the ac-ISHE signal. Unlike the dc voltage, the ac-ISHE signals are expected to suffer from significant transmission losses when they propagate from the sample (Pt/$Ni_{80}Fe_{20}$ stripe) to the detector, i.e., the power meter or the Schottky detector diode. Thus the detected ac ISHE voltages should be lower than the amplitudes one might expect from theory. In order to estimate the amplitude of the ac voltage at the sample it is necessary to consider the characteristic impedances of the Pt/$Ni_{80}Fe_{20}$ (Z_0)-waveguide and the detector ($Z_1 = 50\ \Omega$). For a Pt/$Ni_{80}Fe_{20}$ stripe placed with an insulating layer on top of the waveguide (in-plane configuration) one has a configuration equivalent to a microstrip waveguide. Taking into account sample dimensions and the thickness of the dielectric spacer the characteristic impedance can be calculated as $Z_0 = 480\ \Omega$. From this the transmission into the detector can be computed using the voltage standing wave ratio $T = 1 - \frac{Z_0 - Z_1}{Z_0 + Z_1} = 0.18$. On the other hand when the bilayer wire is placed in the gap (out-of-plane configuration) one has a coplanar waveguide configuration. For the dimensions used in our experiment we expect $Z_0 = 250\ \Omega$ leading to $T = 0.33$. Hence the signal at the detector is reduced due to the impedance mismatch and the original ac-ISHE signal is given by $U^{\mathrm{ac}}_{\mathrm{ISHE}}/T$. In addition a reduction of the measured ac-ISHE signal due to Ohmic losses and imperfect microwave properties of the bonding wires is expected.

Supplementary Note 3

Calculation of inverse spin Hall and inductive signals. For the FM/NM bilayer, the stray field generated by the dynamic magnetization can cause magnetic flux to penetrate the conducting wire loop which is used to pick up the ac-ISHE signal (Faraday's law). Such inductive coupling can result in ac- voltages. The magnitude of this parasitic effect is expected to scale as [3–5]:

$$U_{\mathrm{FMI}} = -i\omega\frac{\mu_0 L d}{2} m_y \eta, \tag{14}$$

where L is the wire length, $d = 10$ nm is the thickness for both FM and NM layers, η ($0 \leq \eta \leq 1$) is a factor which accounts for the fact that only a fraction of the magnetic that flux actually contributes to the inductive voltage, μ_0 is the permeability, and m_y is the dynamic in-plane magnetization component. For a single magnetic layer, both the magnetization and the conducting loop are centered at the same height ($d/2$), and thus one expects no net flux penetrating the wire loop i.e. $\eta = 0$ leading to $U_{\mathrm{FMI}} = 0$. When the ferromagnet is capped by a nonmagnetic metal layer, the center of the conducting loop shifts towards the NM layer by a distance ΔZ:

$$\Delta Z = d\frac{\sigma_{\mathrm{NM}}}{\sigma_{\mathrm{NM}} + \sigma_{\mathrm{FM}}}, \tag{15}$$

Where σ_{NM} and σ_{FM} are the conductivities for the FM and NM layers, respectively. Although it is difficult to calculate the factor η directly, when $\Delta Z \ll w$ (ΔZ is a few nm, and $w = 5$ μm), η is proportional to ΔZ. Consequently, the inductive ac- voltages are expected to scale as

$$U_{\mathrm{FMI}} \sim \frac{\sigma_{\mathrm{NM}}}{\sigma_{\mathrm{NM}} + \sigma_{\mathrm{FM}}}. \tag{16}$$

With σ_{Py}, σ_{Pt}, σ_{Au}, σ_{Cu}, and σ_{Al} equal to 1.3, 3.7, 10.2, 5.1, and 1.4 $\times 10^6$ Sm^{-1} (we determined these values from the resistivities of our samples), we can estimate the ratio of U_{FMI} for the Pt and Au capping layers as:

$$U^{\mathrm{Pt}}_{\mathrm{FMI}} : U^{\mathrm{Au}}_{\mathrm{FMI}} = \frac{\sigma_{\mathrm{Pt}}}{\sigma_{\mathrm{Pt}} + \sigma_{\mathrm{Py}}} : \frac{\sigma_{\mathrm{Au}}}{\sigma_{\mathrm{Au}} + \sigma_{\mathrm{Py}}} = 0.83 : 1 \tag{17}$$

. While for ac-ISHE voltages, the ratio for Pt and Au can be expected to follow the corresponding dc-ISHE signals considering the different susceptibilities [6]:

$$U^{\mathrm{Pt}}_{\mathrm{ISHE-ac}} : U^{\mathrm{Au}}_{\mathrm{ISHE-ac}} = \frac{U^{\mathrm{Pt}}_{\mathrm{ISHE-dc}}}{\chi^{\mathrm{Pt}}_{yz}} : \frac{U^{\mathrm{Au}}_{\mathrm{ISHE-dc}}}{\chi^{\mathrm{Au}}_{yz}} = 12 : 1. \tag{18}$$

Due to the different physical origin of ac-ISHE and inductive signals one expects a 90° phase shift between U_{ISHE} and U_{FMI}. Experimentally we have measured the following ac-voltage amplitudes for the Pt/$Ni_{80}Fe_{20}$ and the Au/$Ni_{80}Fe_{20}$ bilayer (cf. Fig. 3b):

$$U_{\mathrm{ac}}^{\mathrm{Pt}} = 648\ \mu\mathrm{V} = \sqrt{(U_{\mathrm{ISHE-ac}}^{\mathrm{Pt}})^2 + (\mathrm{V}_{\mathrm{FMI}}^{\mathrm{Pt}})^2}, \tag{19}$$

$$U_{\mathrm{ac}}^{\mathrm{Au}} = 94.0\ \mu\mathrm{V} = \sqrt{(U_{\mathrm{ISHE-ac}}^{\mathrm{Au}})^2 + (U_{\mathrm{FMI}}^{\mathrm{Au}})^2}. \tag{20}$$

Taking into account the above ratio for U_{ISHE} and U_{FMI}, they can be calculated for the Pt/$Ni_{80}Fe_{20}$ sample as:

$$U_{\mathrm{ISHE-ac}}^{\mathrm{Pt}} = 645\ \mu V, \tag{21}$$

$$U_{\mathrm{FMI}}^{\mathrm{Pt}} = 63.9\ \mu V, \tag{22}$$

and for the Au/$Ni_{80}Fe_{20}$ sample as,

$$U_{\mathrm{ISHE-ac}}^{\mathrm{Au}} = 53.8\ \mu V, \tag{23}$$

$$U_{\mathrm{FMI}}^{\mathrm{Au}} = 77.1\ \mu V. \tag{24}$$

We can further estimate the inductive ac-voltage for the Cu/$Ni_{80}Fe_{20}$ and Al/$Ni_{80}Fe_{20}$ bilayer samples. In these samples the SHE effect is so small that no dc-ISHE signal was detected and hence also no measurable ac-ISHE is expected. With $\sigma_{\mathrm{Al}} = 1.3 \times 10^6\ \mathrm{Sm}^{-1}$, and noting that the NM=Cu layer is 20 nm thick we can calculate

$$U_{\mathrm{ac}}^{\mathrm{Cu}} = U_{\mathrm{FMI}}^{\mathrm{Al}} = 1.3 U_{\mathrm{FMI}}^{\mathrm{Au}} = 83.1\ \mu V, \tag{25}$$

$$U_{\mathrm{ac}}^{\mathrm{Al}} = U_{\mathrm{FMI}}^{\mathrm{Cu}} = 0.56 U_{\mathrm{FMI}}^{\mathrm{Au}} = 43.1\ \mu V. \tag{26}$$

All of these results are in good agreement with the observed signals cf. Fig. 4(b) of the manuscript.

Supplementary References

---

[1] Tserkovnyak, Y., Brataas, A. & Bauer, G. E. Enhanced gilbert damping in thin ferromagnetic films. *Physical Review Letters* **88**, 117601 (2002).

[2] Mizukami, S., Ando, Y. & Miyazaki, T. Ferromagnetic resonance linewidth for nm/80nife/nm films (nm= cu, ta, pd and pt). *Journal of magnetism and magnetic materials* **226**, 1640–1642 (2001).

[3] Silva, T., Lee, C., Crawford, T. & Rogers, C. Inductive measurement of ultrafast magnetization dynamics in thin-film permalloy. *Journal of Applied Physics* **85**, 7849–7862 (1999).

[4] Weiler, M., Nembach, H. T., Shaw, J. M. & Silva, T. J. Comment on "Detection of Microwave Spin Pumping Using the Inverse Spin Hall Effect". *Preprint at http://arxiv.org/abs/1401.6407* (2014). 1401.6407.

[5] Weiler, M., Shaw, J. M., Nembach, H. T. & Silva, T. J. Phase-sensitive detection of spin pumping via the ac inverse spin Hall effect. *Preprint at http://arxiv.org/abs/1401.6469* (2014). 1401.6469.

[6] Obstbaum, M. *et al.* Inverse spin Hall effect in Ni81Fe19/normal-metal bilayers. *Phys. Rev. B* **89**, 060407 (2014). 1307.2947.
